# “It’s a reasonable gamble”—rural residents’ experience participating in cancer clinical trials at a single rural trial unit

**DOI:** 10.1186/s13063-025-08731-y

**Published:** 2025-02-25

**Authors:** Narelle J. McPhee, Diane Hughes, Eli Ristevski

**Affiliations:** 1 Bendigo Regional Cancer Centre, Bendigo Health, 100 Barnard Street, Bendigo, Bendigo, VIC 3550 Australia; 2 School of Rural Health, Monash University, Bendigo, VIC Australia; 3 School of Allied Health, Faculty of Health Science, Australian Catholic University, Ballarat, VIC Australia; 4 School of Rural Health, Monash University, Warragul, VIC Australia

**Keywords:** Cancer clinical trials, Rural health, Equity, Access

## Abstract

**Background:**

We conducted a qualitative study to examine what factors influence rural-residing people with cancer to participate in cancer clinical trials (CCT) and what factors influence their retention in CCT.

**Methods:**

Purposive sampling was used to recruit participants from a regional cancer centre in Victoria, Australia, to participate in a semi-structured interview. Eligible participants were ≥ 18 years of age at the time of cancer diagnosis, newly consented to a clinical trial (< 1 year) or have been a trial participant for ≥ 1 year, lived in a non-metropolitan area classified within the Monash Modified (MM) Model 2–7 and able to provide informed consent. Thematic analysis was used to analyse the interview data.

**Results:**

Seventeen participants were interviewed; 10 identified as female and seven as male. Participant’s ages ranged from 52 to 77 years, with a median age of 62 years. Eight participants had been on a CCT for ≤ 1 and 10 for ≥ 1 year. Factors that influenced their decision to participate in a CCT included trust and confidence in clinical trial staff, exposure to and trust in the experiences of cancer peers, altruism, low-risk trials and local access to trials. The factors influencing their decision to remain in a CCT included balancing the benefits and burdens of the trial, having no doubts about participating despite knowing the risks and seeing the personal benefits of participating in a CCT.

**Conclusion:**

Our study shows that trust-based relationships, peer support, and altruism encourage rural residents to participate in CCT. To improve access to CCT for rural residents, a multi-faceted approach involving clinicians, health services, trial sponsors and policymakers is needed. These approaches must promote and facilitate the inclusion of diverse populations, prioritise CCT participation, and inform patients of CCT opportunities. We must recognise the knowledge and expertise of rural patients and caregivers and ensure they are involved as co-designers of future CCTs.

## Background

Cancer clinical trials (CCT) inform evidence-based cancer care and allow participants to access cutting-edge treatment [1]. CCT enable participants to receive treatments consistent with standard-of-care guidelines and may improve survival [2]. Despite these benefits, participation rates in CCT are relatively low; in high-income countries, participation rates range between 5 and 14% [3]. There are also known populations that are underrepresented in CCT; rural cancer patients are one such population [3]. Increasing the participation of rural cancer patients in CCT may improve the survival gap between people in rural and metropolitan areas [2]. A study by Unger, Moseley [2] found similar mortality rates for rural residents participating in clinical trials compared to their metropolitan counterparts.

The barriers to CCT participation for people affected by cancer are well documented. A systematic review by Unger, Cook [4] describes participation-related barriers as structural (e.g. availability of a clinical trial), clinical (e.g. trial eligibility criteria), physician-related (e.g. attitude) and patient-related (e.g. attitude). The effect of these barriers varies based on the patient’s demographic and socioeconomic factors. A recent scoping review identified barriers specific to rural populations, including travel and distance, absence of a trial protocol, out-of-pocket expenses, and physician and patient attitudes and knowledge about CCTs [3].

The need to improve access and equity to CCT and care closer to home is well recognised [5–8]. In the Australian context, it was recently identified that 74% of rural residents who participated in a cancer clinical trial had to travel to a metropolitan centre to participate [9]. This highlights the challenges that rural communities face in accessing CCT. Initiatives such as clinical trial networks and tele-trials have been shown to increase the participation of rural cancer patients in CCTs [10–17]. In addition to understanding the barriers to participating in CCT, there is a need to understand what factors can facilitate enrollment into CCT for people in rural areas [8]. Forbes, Shepherd, and Bradford (17) recently examined patient experiences in CCTs, focusing on communication with trial staff overtime at a large metropolitan CCT unit. The authors found effective communication practices were crucial for trial recruitment, retention, and patient satisfaction. The specific experience of rural residents, if included, was not highlighted. There is a paucity of evidence reporting on the experience of rural residents participating in clinical trials locally and how this experience could be utilised to improve rural residents’ participation.

To explore this knowledge gap, we conducted a qualitative study to examine (1) what factors influence rural-residing people with cancer to participate in a CCT and (2) what factors influence the retention of rural-residing people with cancer in CCT.

## Methods

We used a qualitative descriptive design [18] as this methodology provides exploration of an under-reported topic. Participants were recruited from a CCT unit at a large regional-based hospital in the Loddon Mallee Region, Victoria, Australia. The hospital is 150 km (approximately 2 h by car) from the closest specialist metropolitan cancer centre. The Loddon Mallee Region, located in Central and Northwest Victoria, covers 25% of the state’s land area and has a population of 346,668 [19]. Purposive sampling was used to recruit participants between the 24th of February to the 6th of October 2022. Eligibility included; age ≥ 18 years of age at the time of cancer diagnosis, newly consented to a clinical trial (< 1 year) or having been on trial for ≥ 1 year, able to give informed consent, and living in a non-metropolitan area classified within the Monash Modified (MM) Model 2–7 (i.e. rural, regional, remote). The MM Model categorises areas according to geographical remoteness and population size as defined by the Australian Bureau of Statistics [20]. A flyer was shown to potential participants at their oncology appointment by their medical oncologist, and they could express their interest in participating to their medical oncologist or contact the researchers directly. A study team member gave those patients who expressed interest in participating a participant information and consent form (PICF) to read in either paper form or via email. Participants gave their written consent immediately before participating in their semi-structured interview by team member NM. The study was approved by the Health Service Human Research Ethics Committee (LNR/21/BH/77595) and Monash University Human Research Ethics Committee (31,406).

Semi-structured interviews were used to collect the data and were conducted by author NM either in person or via videoconference. In-person interviews were conducted at the cancer centre before or after the next scheduled CCT appointment to reduce participant travel burden. Interviews were conducted between 1st April and 31st October 2022. Two sets of interview questions were developed: one for recently consented clinical trial participants and one for participants who had been on a clinical trial for longer. The research team initially created the interview questions that were then refined/vetted by two local Cancer Consumer Advisory Group representatives. The complete interview guide is provided as supplementary material. Interviews were audio recorded, transcribed verbatim by a professional medical transcription service, and imported into NVIVO 12 Software (QSR International Pty Ltd, 2018) for coding and analysis. As part of the member-checking process, participants were posted a summary of their interview responses to check the researchers had interpreted their views and experiences accurately; no participants replied to request any changes to their interview data.

Thematic analysis according to Braun and Clarke’s method [21] was used to analyse the data and was conducted through six phases. Phases 1 and 2 initially involved one researcher (DH) reading the interview transcripts, listening to the audio recordings several times and making notes on initial thoughts and observations noted in the data. In phase 2, an initial code (or name) was attributed to phrases, sentences or paragraphs based on the semantic content. Codes were then either expanded in number and complexity or reduced; earlier coding was re-visited to ensure coding consistency across the entire data analysis period. In phases 3–6, all researchers met to discuss and review the coding and refine the data into themes [21]. In phase 3, initial themes from patterns observed in the codes were generated. These themes were further refined in phase 4 by reducing, expanding or merging themes (thus patterned) and examining how the themes contributed to answering the research questions. In phase 5, we used concept maps in NVIVO 12 Software to finalise the themes and generate the final theme and sub-theme names. Phase 6 involved writing up and interpreting the findings of the analysis within the context of the peer-reviewed literature. To maintain participant confidentiality, pseudonyms were allocated to participants by an internet search for the ‘top names of 1900’s’ using the US Government website [22]. The research team comprises 20 years of experience in health, research, and service improvement and diverse social and professional backgrounds. All authors identify as female. NM and DH are of Caucasian background, and ER comes from a culturally and linguistically diverse (CALD) background. All authors live and work in rural areas. NM is the Cancer Research Manager, and DH as a Clinical Trial Coordinator at the Cancer Centre. NM does not work directly with trial participants and, therefore, is not known to the participants before the interviews. ER works as an academic at the University School of Rural Health. All researchers have a focus on rural access to quality health care. The researchers also have personal connections with family and friends who have been diagnosed with and treated for cancer.

## Results

### Participant overview

Seventeen people participated in our study; ten were female, and seven were male. Participants’ ages ranged from 52 to 77 years, with a median of 62 years. Educational attainment included high school (*n* = 8), university (*n* = 4) and other (e.g. diploma, certificate) (*n* = 5). Most participants were not working (*n* = 11), and three were primary carers. Most participants had been diagnosed with colorectal (*n* = 7) or breast cancer (*n* = 5). Eleven participants had locally advanced disease and five had metastatic disease. One participant had been on two clinical trials, initially for locally advanced disease and then later for metastatic disease. There was a balance of newly consented participants to a clinical trial (≤ 1 year) (n = 8) and those who had been on trial for ≥ 1 year (*n* = 10). All participants spoke English as their first language. The mean time on a CCT was 20 months. Of the CCT, five were curative, three were palliative, and one was supportive care (Table 1). Interview duration ranged from 16 to 49 min, and of the 17 interviews, 15 were conducted in person and two by video-conference. All patients attended their interviews unaccompanied. Two major themes were identified in the data: (1) deciding to participate in a CCT and (2) balancing the benefits, risks and burden of CCT.

**Table 1:** Participant demographic and clinical characteristics

Participant pseudonym	Gender	Age^1^	Highest education	Work status^1^	Tumour		Trial intent	Length of time on clinical trial^2^	
					**Stage**	**Type**		**Years**	**Months**
Arthur	Male	76	University degree	Working	Metastatic	Melanoma	Palliative	0	10
Bernard	Male	61	Industry certificate	Not working	Metastatic	Melanoma	Palliative	1	0
Beverly	Female	77	≥ Year 10 high school	Working	Locally advanced	Breast	Curative	2	11
Carolyn	Female	45	University degree	Working	Locally advanced	Breast	Curative	0	5
Clarence	Male	69	≥ Year 10 high school	Not working	Locally advanced	Colorectal	Supportive care	1	3
Gladys	Female	69	≥ Year 10 high school	Not working	Locally advanced	Breast	Curative	2	8
Joel	Male	61	Industry certificate	Working	Metastatic	Melanoma	Palliative	1	3
Kathleen	Female	61	≤ Year 10 high school	Not working	Locally advanced	Colorectal	Curative	3	7
					Metastatic	Colorectal	Palliative	0	6
Lena	Female	61	University degree	Working	Locally advanced	Colorectal	Curative	0	3
Marilyn	Female	57	University degree	Working	Locally advanced	Colorectal	Curative	1	10
Marion	Female	77	Industry certificate	Not working	Metastatic	Genitourinary	Palliative	0	4
Perry	Male	58	≥ Year 10 high school	Not working	Locally advanced	Lung	Curative	1	5
Russell	Male	52	≥ Year 10 high school	Not working	Metastatic	Colorectal	Palliative	0	11
Sadie	Female	69	Industry certificate	Not working	Locally advanced	Breast	Curative	0	4
Sidney	Male	67	≥ Year 10 high school	Not working	Locally advanced	Colorectal	Curative	4	2
Theresa	Female	62	≥ Year 10 high school	Not working	Locally advanced	Breast	Curative	2	8
Wilma	Female	77	Industry certificate	Not working	Locally advanced	Colorectal	Curative	3	8

^1^ At the time of interview

^2^ Rounded up to the nearest month

### Deciding to participate in a CCT

Within the theme ‘deciding to participate in a clinical trial’, the following sub-themes were identified.

- Trust and confidence in clinical trials staff
- Exposure to and trust in the experiences of cancer peers
- Altruism
- Local access to trials
- Low-risk trials

#### Trust and confidence in clinical trials staff

*Trust and confidence* in the advice of the medical oncologist to consider a CCT, as well as confidence in the coordination skills of clinical trials staff (research nurses and/or CCT study coordinators), were important in participants’ decision to participate in a CCT. Participants reported there was a lot of information given to them about the clinical trial processes and treatments they would receive, and the CCT PICFs were difficult to understand. *Theresa responded to the interviewer that said, “….there would have been… a twenty-five-page consent form that you would have read and signed.” I will admit that I really didn’t read it word for word. A lot of it was over my head.*

Participants placed deep trust in the medical oncologist explaining the clinical trial and helping them to understand all the information about consent and treatments. Perry: “… as much information that I could absorb, I was given … how it was explained to me was quite clear, and I understood everything.” Russell: “The oncologist was very good at explaining everything for me, and I was just comfortable with his advice, I suppose, from that perspective.”

Participant’s deep trust in the medical oncologist was also expressed in their interpersonal qualities and communication skills; Russell and Arthur used the term “faith”, and Beverly felt “very comfortable in discussing anything with him”. Lena described her medical oncologist as “very caring” and “very goodat discussing things”. Joel’s decision was based on the expertise of his medical oncologist; “I didn’t really think I had too much choice of where else to go, but at the end of the day, and I’ve said it in my business forever, if you pay an expert to do a job, well you listen to him….”

Participant’s trust and confidence in the clinical and coordination skills of and rapport with clinical trials staff was also apparent. Beverly describes the trial coordinator as “bright and breezy and bubble and very good at what she does”. Carolyn stated, “feeling like I’m not a number … I’m remembered,” and more broadly, that she “Feels loved…[during] the whole process”. Marion described clinical trials staff as “very professional and they do what they do very well”. Perry found the care he received to be “pleasurable to a point” and has kept returning for follow-up care despite moving more than 1600 km away.

#### Trust in the experiences of cancer peers

Trust in the experiences of, and connections with, other people who had already been on a CCT was a factor in helping participants decide whether to participate in a CCT. Beverly disclosed she discussed the idea of the clinical trial with her family, particularly her husband, but did not find that helped her to decide. “..you know the decision was always going to be mine and mine alone, no matter what.” Lena said a friend with cancer participated in a CCT that “extended his life by over five years through that”, and that got her interested in CCT. Russell mentioned a friend with cancer from his hometown recommended a medical oncologist to speak with “about trials and things”. Speaking with his cousin, who is on a haematology CCT, gave Russell more understanding and confidence to consent to a CCT. Carolyn’s comments on her mother’s CCT experience also demonstrate this trust in other cancer patient’s experience of clinical trials; “I think that really sat with me and thinking you know what, if my mum was brave enough back then when there was nothing…. you had to go into a library to read anything. If she could have done it, there’s no reason why I can’t do it, and there’s so much information for me to read.”

#### Altruism

Altruism was also a factor in deciding to participate and stay in a CCT. Altruism was described in terms of benefits for the medical oncologist’s own learning and clinical practice, medical knowledge as a whole (scientific knowledge), and other people with cancer. Russell stated: “… if the oncologists and the researchers, can use the information that they get from me to help somebody else … that’s what you’re doing it for, to be honest.”

Marilyn felt *“useful”* and that she was contributing to “knowledge” and helping the next patients. Sadie wanted to contribute to scientific knowledge; “I felt you can’t always contribute personally a lot to thefurtherment of science and medicine, and I thought, this is a way I can contribute… it’s only my time.” Beverly was thinking of others; “I was pretty committed right from the word go because I thought some good will come out of this …if not for me but maybe for others.”

Some participants recognised that other people with cancer had participated in earlier clinical trials that contributed to the treatment they received, Gladys expressed: “Oh, yeah but people have been generous for us too.” Kathleen noted the same idea “I look stuff up on the internet to find out information, if somebody else hadn’t…been through that, how could I find that out?”, as did Clarence: “I mean the total care I’m getting now is because of what was done years ago.”

#### Local access to trials

Access to a CCT in a local rural hospital was important to some participants, and they valued the option to choose between a rural and metropolitan location. Beverly’s General Practitioner (GP) gave her the option of going to a metropolitan or rural-based CCT. Beverly decided on a rural-based trial and was “thrilled” as she felt “if I can have this treatment in a place that I’m more comfortable,that’s what I’m going to do.” Joel was emphatic on preferring the rural cancer centre over the metropolitan alternatives; “I’m lucky that I can come to a regional city because I keep saying to people, if I had to go to the state capital city as often as I come to a regional city, I’d bloody want to die.”

Participants did not feel they were missing out on highly specialised metropolitan medical care as they knew this could be linked in if required. Arthur stated: “I just think I’ve been very fortunate to have, the medical services close at hand and, then, even if the -, the expertise isn’t all here, at least the link to the, metropolitan specialist cancer hospital or wherever, can be made very easily.”

### Balancing the benefits and burden of the trial

Within the second major theme, “balancing the benefitsand burden of the trial”, the following sub-themes were identified:

- No doubts
- It is a reasonable gamble
- Personal benefits
- Burden

#### No doubts

Despite the uncertainty about their health, participants were emphatic about having no doubts about participating in the CCT, exemplified by Theresa’s answer when asked if she had any doubts about participating in a CCT: “No, no, not at all”. Participants indicated they were comfortable with their medical information and CCT data being used and shared with the trial sponsor to help answer the clinical trial objectives. Russell observed a large section of the CCT PICF was devoted to how data would be shared but stated, “To be honest, I don’t care about that” and “…you could share it with the world. It wouldn’t worry me.” Kathleen sees her data as “past history” and if it “might help the next person, go for it.”

#### It is a reasonable gamble

The absence of doubt exists within the context of uncertainty about what would occur on the CCT., as expressed by Bernard: “Oh, I had no idea what was going to happen…I just thought well it will work or it won’t.” Other participants, such as Marion, when thinking about her cancer, had to “weigh up the fact that I know that it’s rare and I know it’s aggressive”, but her understanding of some trial results indicated “people responded in some way. So it was a reasonable gamble.” Further, when asked about her clinical results, she said, “you had no idea it was going to happen.” Marion’s awareness of another CCT using the same treatment for a different cancer with promising results made her feel her decision was “a bit of a no-brainer.”

Marilyn felt she was handing over her care and explained she thinks trust is: “really important” in handing over her care, and she was prepared to do this;“…because I’m a nurse, I want to have some input into my care, and I can’t really because I’m the patient. So, I’ve just given all of that to someone else…”.

Participants knew the risks and judged their own circumstances despite the uncertain outcomes. Bernard stated; “…., I didn’t have anything to lose because, well, if I didn’t go ahead with it, I probably wouldn’t be here talking to you anyway.” In many instances, participants understand they have limited treatment options and were prepared to take a risk, as Russell expressed; “I think when you’re you’re not given a really good outlook, and the option is there to be on trials and that, I was happy to be involved in it.”

#### Personal benefits

Participants also saw a direct personal benefit to participating in a CCT in that they received more attention from more staff involved in the clinical trial. Arthur noticed the “precision” of clinical trial treatment processes, which he finds “reassuring”. Sidney described this extra monitoring as “an insurance policy” because “I get tested more often…and there’s a bit more follow-up”. Clarence felt he had “contact with professionals on a more regular basis”, an idea echoed by Wilma: “I think I’ve had above and beyond for what I would have normally had.” Russell felt “like I’m getting a little bit of VIP treatment to be honest.” Participants also expressed hope for personal benefit from participating in the trial, Sadie: “…I guess I was hoping that I would be put on the new drug.”

#### Burden

Participating in a clinical trial was not always described as a burden; instead, some participants saw their cancer as the burden. Kathleen said, “there’s no difference; …the trials teams is in the background to my care; they’re just…taking the information away”. Beverly had to make an hour-long return trip every three months for a blood test at a pathology service to meet the CCT requirements. Beverly was pragmatic in describing this extra travel; “it was just an extra trip you had to do”. Carolyn felt unsure if she was doing enough: “I feel like taking a tablet and writing out a diary, is that it?”.

There were other views, though, as Sadie observed that being a clinical trial participant “requires you to have discretionary time” and that this could be “a potential problem and would…prevent people from saying, oh yes, I’ll be in this”. Theresa mentioned being taken aback by the extensive time commitment required” the reality of it…in the beginning was oh my gosh…this is what I had to do” even after she confirmed staff had made the time commitment very clear.

Participants were also aware they could withdraw from the CCT if the burden became too much, but, as mentioned by Beverly; “You know, the option’s always there. But it was never in my thinking that I wouldn’t complete it.” Retention on the clinical trial is easier for participants if they remember the reasons they enrolled on the trial, as expressed by Carolyn; “You want to go on a trial, remember you’re not just doing it for you, you’re doing it for others. So once you say yes, and it gets hard, remember that this is not just about you…it’s not a short-term journey that you think, now I just want to get off because I can’t be bothered.”

## Discussion

We conducted a qualitative study to explore the unique experiences of rural-residing participants who enrolled in CCTs at a cancer centre in Australia located in a rural area. Participants in our study identified trust and confidence in their medical oncologist and clinical trial staff as important factors influencing their decision to participate in a CCT. Furthermore, trust in the experience of and connections with cancer peers who had previously participated in a clinical trial was another important factor. Participation was viewed as a reasonable risk. Study participants expressed no doubts about participating in a trial and described their decision to join as an obvious one. Additional clinical appointments and associated travel were a burden for some participants, but for other participants, the extra care and treatment were viewed as providing personal benefits. Altruism and willingness to help other cancer patients and their medical oncologists develop their own clinical research experience positively influenced decision-making.

Our finding of trust as an essential component of deciding to participate in a CCT is consistent with other qualitative studies exploring CCT participation in metropolitan-residing CCT participants [23, 24], rural-residing CCT participants [25], and rural-residing people with cancer who did not participate in CCT [26]. Fiduciary trust in a physician reflects the patient’s belief that the physician will act in their best interests and not exploit their vulnerability [27]. Consistent with our study, Coyne, Demian-Popescu [26] reported that rural-residing people with cancer who have a strong trust in their physicians often rely entirely on their doctor’s treatment recommendations. Receiving cancer care at a local cancer centre may have enhanced participants’ trust in their treating physician, especially if they had previously received treatment from the treating physician who also discussed clinical trial participation. Receiving care locally can reduce the burden of travel and difficulty attending appointments. It is interesting to observe that the participants in our study attended the interview unaccompanied. This perhaps indicates that participants were less dependent on family members or friends to attend appointments and, therefore, were not reliant on others to decide to participate in a clinical trial. Family members’ influence on the clinical trial decision-making process was less significant for our participants.

We found peers were a trusted source of information, increasing participant’s confidence and familiarity with the clinical trial process and influencing their decision to participate in a trial more than family members. Peer support is a well-recognized strategy used in cancer care to support patients’ physical, mental, and social well-being through education and social interaction [28]. For example, Cancer Council Victoria trains past clinical trial participants to provide peer support via the telephone to help individuals considering participating in a clinical trial and would like to discuss the experience with someone [29]. Peer support has been used in culturally, linguistically, and racial and ethnic minorities to aid clinical trial participation [30]. Culturally appropriate patient navigators have also been shown to increase clinical trial participation for rural residents [31, 32].

Consistent with our study, altruism is a critical factor in the decision to participate in clinical trials [33]. Motivation to participate in CCT results from participants feeling that the trial results will benefit others who come after them. An interesting finding in our study was the acknowledgement that the current standard of care treatments resulted from past CCT participants. This finding is consistent with a Swedish study, where clinical trial participants acknowledged previous study participants’ contributions, which motivated them to participate in clinical trials [23]. Massett, Dilts [34] described that acknowledging past participation in clinical trials helps normalise the process of clinical trial participation. This is a positive message that increases interest in clinical trials. Naidoo, Nguyen [35] conducted a systematic review of qualitative studies to explore the experiences of adult patients who participated in randomised controlled trials (RCTs). Consistent with our results, the benefits of clinical trial participation in this study included altruism, satisfaction resulting from feeling “useful”, and the benefit of close monitoring [35].

Our results highlighted the importance of local access to CCT so that participants received care closer to home. Receiving care closer to home reduces the time and costs associated with clinical trial participation. Travel and distance remain barriers to CCT participation, as acknowledged by physicians [36–38] and cancer patients [26, 38–40]. Strategies to overcome these barriers include using tele-trials and clinical trial networks [3]. Tele-trials use telehealth technology to connect a primary trial site to one or more satellite sites [41]. Clinical trial networks are formed through partnerships, and their members may include academic medical centres, community groups, and rural health services. Clinical trial networks may also have financial support to establish infrastructure and fund dedicated clinical trial positions to support new trial sites [10, 12, 14, 42, 43].

Local access to clinical trials reduces the costs associated with participation. Compared to usual care, clinical trial participation leads to more visits to a cancer care provider, placing an additional financial burden on participants [6]. This burden is further compounded for those rural residents who must travel to metropolitan centres for clinical trials. Financial toxicity is worse for those clinical trial participants living greater than 100 miles away from their clinical trial unit and those with lower socioeconomic status [44, 45]. Staying close to family and consistency of care were valued by participants in a regionally conducted teletrial [46]. Patients who travel away from home for clinical trials may do so due to the lack of opportunities for local trial participation. Patients may feel obligated to endure the cost and burden of travelling to a metropolitan area to participate in a CCT [46].

A factor in consent for some participants was the lower risk profile of the clinical trial they were offered. CCTs with lower risk, such as trials comparing existing standards of care, were identified as easier to consent to by the participants in our study. Consistent with Prang, Karanatsios [33], our participants were more comfortable with trials using known (regulatory-approved) drugs compared with novel therapies. While RCTs are seen as the gold standard of high-quality research [47], Prang, Karanatsios [33] explored the feasibility and acceptability of registry-based RCT (RRCT) as an alternative to increasing participation in clinical trials. A RRCT combines the methodology of a RCT with the practical benefits of data from registry studies, and they are cost-effective and pragmatic in answering clinical questions [48]. RRCTs offer a more straightforward study design and are less demanding to conduct or participate in than RCTs [33]. They provide an alternative for RCT that may assist in increasing clinical trial participation outside the metropolitan area.

Clinical trials were seen as a last resort for some participants in our study. This means that all lines of standard-of-care treatment have been utilised, and no other treatment options were available. According to Krieger [49] study on rural women who had experienced cancer or cared for someone with cancer, the perception of clinical trials as a final option has a greater negative impact on their participation in prevention trials or new therapies for highly treatable cancers, compared to trials for cancers with poor prognoses.

In our study, participants were not concerned about sharing their medical information and saw this as beneficial to meet trial objectives and a meaningful way to benefit others. They did not identify sharing data as a risk. This is consistent with the findings of a cross-sectional study of 677 cancer patients/survivors by Franklin, Nichols [50], where 71% of participants were willing to share de-identified medical data, and most participants (88%) were motivated by altruism.

This is the first Australian study of rural clinical trial participants attending a clinical trial unit outside a metropolitan area. The results of this study have broader applicability than oncology. Given the benefit of clinical trial participation and the underrepresentation of rural populations, improving access to all types of clinical trials is important. Improved clinical trial access will increase the generalizability of clinical trial results and give rural populations the same access to treatment that metropolitan populations experience. Understanding what is important to rural clinical trial participants will help overcome some barriers to clinical trial participation more broadly.

The semi-structured interviews encouraged participants to share their experiences in their own words. The member check process added rigour to the interpretation of the data. A limitation of our study is the lack of participants from a CALD background and Australian First Nations peoples. By having a predominantly Caucasian participant group, the study’s findings may not accurately represent the experiences or responses of CALD and Australian First Nations peoples. Without additional research, conclusions drawn from a Caucasian-centric sample cannot be reliably extrapolated to other cultural groups CALD and Australian First Nations peoples are known to be underrepresented participants in clinical trials [51, 52]. There is well-documented evidence of high levels of mistrust of health care professionals and health care services for ethnic and racially diverse populations [53, 54]. Lack of diverse representation in clinical trials contributes to ongoing health disparities and potentially less effective healthcare interventions for minority populations. Another limitation is that the study was conducted at one clinical trial unit. Participants in our study may not represent all clinical trial participants living outside the metropolitan areas. Those who agreed to participate in our study were clinical trial participants who in most cases had a positive clinical trial experience and were willing to share, which introduces a potential bias.

## Conclusion

Our study provides insight into the experiences of CCT participants from rural areas. Trust-based relationships with clinical trial staff, cancer-peers support, and altruism are critical factors that facilitate CCT participation. Under-representation of rural residents in CCT could lead to missed opportunities for cutting-edge treatment provided by clinical trials, as well as missed opportunities to contribute to future cancer care. A multi-faceted approach is required to improve rural residents’ access to CCT. Equity of access is a shared responsibility of policymakers, trial sponsors, health services and clinicians. Cancer strategy and trial sponsors should promote and facilitate the inclusion of diverse populations. At the same time, health services must prioritise CCT participation by providing local opportunities and supporting clinicians to be active in clinical trials. Clinicians must be aware of CCT opportunities and discuss them with their patients. The lived experiences of rural patients and caregivers in CCTs have not been adequately recognised or utilised. Our research has demonstrated the willingness of rural residents to participate in CCT. Therefore, it is essential to recognise the knowledge and expertise of these individuals and caregivers and place them as co-designers of future CCTs. Their knowledge and experience can help guide and benefit future CCT participants and contribute to expanding CCT access in rural areas.

## Data Availability

The datasets used and/or analysed during the current study are available from the corresponding author on reasonable request.

## Abbreviations

CCT: Cancer clinical trial
CALD: Culturally and linguistically diverse
RCT: Randomised controlled trials
RCCT: Registry-based randomised controlled trials
